# Systemic Treatment of Vitreous Inflammation

**DOI:** 10.1155/2012/936721

**Published:** 2012-09-16

**Authors:** John B. Christoforidis, Susie Chang, Angela Jiang, Jillian Wang, Colleen M. Cebulla

**Affiliations:** Retina Division, Department of Ophthalmology, Wexner Medical Center at The Ohio State University, 915 Olentangy River Road, Suite 5000, Columbus, OH 43212, USA

## Abstract

Non infectious vitreous inflammation is often vision threatening and can be associated with potentially life-threatening systemic conditions. Treatment is often challenging as it involves systemic medications that can be associated with adverse effects. The classes of drugs are ever expanding and include corticosteroids, antimetabolites, alkylating agents, T-cell and calcineurin agents, biologic agents, and interferons. Each class of systemic therapy for non-infectious vitreous inflammation is reviewed. We discuss the mechanisms of action, usual clinical dosages, the specific conditions that are treated, the adverse effects, and usual course of treatment for each class of therapy.

## 1. Introduction

Vitreous inflammation is a hallmark of posterior uveitis which accounts for 9–38% of uveitis cases [1]. The sequelae of posterior inflammation include visual loss from vitreous opacities, cystoid macular edema, serous retinal detachment, retinal ischemia, neovascularization, retinal pigment epithelium (RPE) changes, and subretinal fibrosis, as well as glaucoma and cataract. Vitreous inflammation may be infectious in origin as in cases of toxoplasmosis, syphilis, *Bartonella*, or infectious endophthalmitis. Many vision and potentially life-threatening causes of vitreous inflammation are noninfectious in origin and include sarcoidosis, Vogt-Koyanagi-Harada (VKH), Behçet's disease, sympathetic ophthalmia, intermediate uveitis, Wegener's granulomatosis and systemic lupus erythematosus (SLE). This paper will focus on systemic treatment of non-infectious causes of vitreous inflammation. 

## 2. Corticosteroids

Corticosteroids are the primary treatment for non-infectious uveitis. Most treatments commence with the use of topical drops or ointments. However, penetration to the posterior segment is limited [2]. Higher local concentrations into both the anterior and posterior segments without significant systemic side effects are achieved by injecting or implanting steroid compounds, such as triamcinolone acetonide, either periocularly or intravitreally [3]. Intravitreal delivery can also be achieved with placement of slow-release devices such as Ozurdex and fluocinolone implants. Although these local treatments are often efficacious, inflammation may recur as the intravitreal steroid concentration decreases. While these local delivery routes have minimal systemic consequences, local side effects such as increased intraocular pressure and glaucoma, cataract, ptosis, and rarely herpetic retinitis can occur. 

For patients with uveitis who do not respond adequately to local forms of steroid treatment or have severe disease, systemic steroid therapy is required to gain control of vitreous inflammation [4]. Oral prednisone is typically the first systemic therapeutic agent used at a dose of about 1 mg/kg/day followed by gradual tapering [5]. Patients on oral corticosteroid therapy are monitored for both response to treatment and adverse effects. Potential complications resulting from systemic steroid therapy include increased intraocular pressure and cataract formation, osteoporosis, hyperglycemia, aseptic bone necrosis, gastrointestinal ulcers, pancreatitis, myopathy, psychosis, delayed wound healing, Cushingoid features, secondary infection and reactivation of latent herpes simplex or tuberculosis. In successful systemic corticosteroid therapy, inflammation subsides and steroids are slowly tapered. If inflammation recurs during tapering, a higher corticosteroid dosage is instituted until inflammation resolves followed by tapering. If reactivation occurs and inflammation persists for over 4 weeks with therapy, or if the patient develops systemic adverse effects, adding locally delivered steroids and/or systemic immunosuppressive therapy should be considered [6]. 

## 3. Immunosuppressive Medications

Systemic immunosuppressive treatments are critical to adequately manage certain underlying causes of inflammation (e.g., Wegener's granulomatosis) and are used as "steroid-sparing" agents when long-term treatment is indicated. Immunosuppressive therapy can replace or supplement corticosteroid therapy. More importantly, early immunosuppressive therapy is helpful in reducing blindness in conditions such as Behçet's disease, VKH, sympathetic ophthalmia, Wegener's granulomatosis, juvenile idiopathic arthritis-associated uveitis, rheumatoid necrotizing scleritis, and ocular cicatricial pemphigoid [6]. Table 1 lists uveitis conditions for which immunosuppressive chemotherapy is indicated. 

Immunosuppressive agents are classified generally as antimetabolites, alkylating agents, T-cell inhibitors/calcineurin inhibitors, and biologic agents. Ophthalmologic conditions for which immunosuppressive agents are used are summarized in Table 2. Individual therapeutic agents within each class are discussed below.

### 3.1. Antimetabolites

#### 3.1.1. Methotrexate

Methotrexate is a folic acid analog that competitively binds with and inhibits dihydrofolate reductase, thereby, reducing production of thymidylate and purine which are essential for DNA replication [7].Highly metabolic inflammatory mediator cells such as leukocytes are suppressed thereby reducing inflammation [8]. Folinic acid supplementation is used with high doses of methotrexate to protect more slowly dividing cells by restoring thymidylate and purine biosynthesis [7].

Methotrexate can be administered orally, subcutaneously, intramuscularly or intravenously at doses ranging between 7.5 to 25 mg per week. It has been reported to reduce vitreous inflammation as well as other inflammatory conditions such as vasculitis, scleritis, anterior uveitis, orbital pseudotumor and sarcoidosis [9–14]. Three to eight weeks are required for the anti-inflammatory effects of methotrexate to take full effect. 

Methotrexate is commonly used as a steroid-sparing agent, allowing steroids to be tapered. Its side effect profile is preferable to that of high-dose steroids for long-term treatment and includes fatigue, nausea, vomiting and anorexia (5–25%), which usually improve following dosage reduction [15]. Less common but serious side effects include hepatotoxicity (15%), bone marrow suppression, cutaneous vasculitis and urticaria [15]. Methotrexate is a teratogen and is contraindicated for use during pregnancy [7]. Laboratory monitoring for side-effects must be performed during methotrexate therapy. Baseline labs include complete blood count (CBC), serum chemistry, blood urea nitrogen, serum creatinine, liver function tests (LFTs), urinalysis and pregnancy test. Maintenance laboratory testing is conducted at 4-week intervals and consists of CBC and LFTs. The methotrexate dose is reduced if liver enzymes double on 2 subsequent measurements and is discontinued if the liver enzymes remain elevated after dose reduction [16]. Therapy is continued for 2 years following ocular quiescence to avoid recurrence of inflammation [17].

#### 3.1.2. Azathioprine

Azathioprine (Imuran, GlaxoSmithKline, London, UK) is an imidazolyl derivative that metabolizes to thioinosine-5-phosphate, a purine analog that interferes with DNA and RNA replication and transcription. It suppresses lymphocyte proliferation and antibody production [18]. It also suppresses natural killer cells and the delayed type hypersensitivity reaction [19]. Azathioprine has been effective in the treatment of serpiginous choroiditis, multifocal choroiditis, panuveitis, ocular cicatricial pemphigoid, and juvenile idiopathic arthritis [20–22]. 

Azathioprine is administered orally at an initial dose of 2 to 3 mg/kg/day and then adjusted based on clinical response and adverse effects. The most common adverse effect is gastrointestinal upset followed by hepatotoxicity, bone marrow suppression (leucopenia and thrombocytopenia), alopecia and pancreatitis [19, 23].Baseline labs should include CBC and LFTs. In addition, Foster and Vitale recommend obtaining blood thiopurine methyltransferase enzyme activity levels at baseline and to withhold treatment if the enzyme activity is low or absent [24]. CBC and liver function tests are repeated at 4 to 6 week intervals during treatment. Treatment dose should be decreased in cases of mild abnormalities and temporarily discontinued and resumed at lower doses in the presence of major abnormalities [25]. Treatment is continued for 2 years following ocular quiescence [24].

#### 3.1.3. Mycophenolate Mofetil

Mycophenolate mofetil (MMF) (CellCept, Roche, Basel, Switzerland) is a reversible inhibitor of the enzyme inosine monophosphate dehydrogenase that is involved in guanosine nucleotide synthesis. It disrupts DNA synthesis that is used by B and T cells for purine synthesis [26]. MMF also interferes with cellular adhesion to vascular endothelium and disrupts lymphocytic chemotaxis [27]. MMF has been found to be effective as monotherapy in the treatment of chronic ocular inflammatory disease [28]. It has also been found to be effective in combination with cyclosporine and methotrexate for the treatment of scleritis and uveitis [29].

Mycophenolate mofetil is administered as an initial dose of 500 mg twice a day and adjusted after monitoring for side effects. The dose is increased to 1 g twice a day if it is well tolerated [24]. Adverse effects are most commonly gastrointestinal including nausea, vomiting and diarrhea. Less frequent side effects include leukopenia, lymphocytopenia, and hepatotoxicity [7]. CBC and LFTsshould be obtained at baseline and followed with weekly CBC for the first 4 weeks, twice monthly for 2 months and monthly thereafter. Liver function tests are obtained monthly for the duration of treatment [30]. Treatment is continued for 2 years following ocular quiescence [24].

#### 3.1.4. Leflunomide

Leflunomide (Arava, Sanofi-Aventis) inhibits the enzyme dihydro-orotate dehydrogenase which is involved in pyrimidine synthesis. Consequently, it interferes with B and T cell proliferation and suppresses the inflammatory response [31]. Suppression of tyrosine kinase and possibly cyclo-oxygenase and histamine release may further potentiate its anti-inflammatory effects [32, 33]. Although ocular use of leflunomide is limited, it has shown promise in the treatment of ocular inflammation associated with sarcoidosis [34]. 

Leflunomide therapy for systemic conditions such as rheumatoid and psoriatic arthritis is typically administered orally with a loading dose of 100 mg followed by 10 to 20 mg daily. The loading dose is necessary because of the long plasma half-life of leflunomide (15–18 days) [35]. The most serious adverse effect is hepatotoxicity which can range from jaundice to fulminant hepatitis. Other adverse effects include headache, paresthesias, leucopenia, anemia, thrombocytopenia and interstitial lung disease [36]. Concurrent use with methotrexate is discouraged because of potential hepatotoxicity. Patients should have baseline CBC and LFTs every 2 weeks for the first 6 months and then every 8 weeks following commencement of treatment. Leflunomide is teratogenic and contraindicated for use during pregnancy [35]. The optimal duration of therapy is currently not certain.

### 3.2. Alkylating Agents

#### 3.2.1. Cyclophosphamide

Cyclophosphamide (Cytoxan, Bristol-Myers Squibb) is an alkylating agent derived from mustard gas. It is cytotoxic to rapidly dividing cells such as T and B lymphocytes by alkylating DNA bases and disrupting DNA cross-linking, and antibody production and delayed-type hypersensitivity is suppressed [37]. Cyclophosphamide has been found to be effective in the treatment of ocular inflammation associated with Wegener's granulomatosis, polyarteritis nodosa and Behcet's disease. It is also used to treat bilateral Mooren ulcer and severe ocular cicatricial pemphigoid and scleritis secondary to rheumatoid arthritis and relapsing polychondritis [38–43].

Cyclophosphamide is preferably administered intravenously rather than orally as induction is more rapidly achieved and bladder exposure is reduced [44]. It is initially administered intravenously at a dose of 1 g/m^2^ and adjusted based on the response, serial CBCs and the presence of any adverse events [30]. Treatment is commenced every 2 weeks and once stabilized treatment intervals are reduced to 3 to 4 weeks.Adverse effects are reversible bone marrow suppression, hemorrhagic cystitis, secondary cancers such as bladder cancer and acute myeloid leukemia, testicular atrophy and ovarian suppression. Cyclophosphamide is teratogenic and contraindicated in pregnancy. Baseline CBC with platelets, liver function tests and urinalysis are obtained. CBCs and urinalysis are repeated weekly initially and all labs are repeated monthly when the blood counts are stabilized. The dose is lowered if there is mild bone marrow suppression and interrupted and restarted at a lower dose if there is severe bone marrow suppression. The dose is discontinued if there is hematuria and consultation with urology is recommended if persistent after 3 weeks [30]. Treatment is continued for 1 year following ocular quiescence [1]. 

#### 3.2.2. Chlorambucil

Chlorambucil (Leukeran, GlaxoSmithKline, London, UK) is an alkylating agent derived from nitrogen mustard. It causes cross linking within DNA strands interfering with DNA replication and transcription [45]. Chlorambucil has been used to treat Behçet's disease, sympathetic ophthalmia and serpiginous choroiditis [46, 47].

Chlorambucil is administered orally as it does not have the deleterious bladder effects seen with cyclophosphamide. Initial dosing is typically 0.1 mg/kg/day with incremental adjustments based on response, clinical labs and adverse effects to a maximum of 12 mg daily. Treatment is continued for 1 year following ocular quiescence [24]. Another treatment algorithm is short-term high-dose oral therapy for 3 to 6 months [30]. Adverse events related to chlorambucil include reversible myelosuppression, bone marrow aplasia, male sterility, amenorrhea, gastrointestinal distress, hepatotoxicity, central nervous system effects such as seizures, secondary cancers, and reactivation of latent herpes simplex virus [30, 48, 49]. Baseline labs include CBC with differential and LFTs. CBC is followed weekly initially and monthly along with LFTs once the dose is stabilized. It is especially important to monitor these patients for myelosuppression since the effect of chlorambucil on the bone marrow is cumulative [30]. 

### 3.3. T-Cell Inhibitors/Calcineurin Inhibitors

#### 3.3.1. Cyclosporine

Cyclosporine (Neoral, Novartis) is a fungal byproduct that binds cyclophilin and calcineurin and inhibits T lymphocytes and their ability to produce lymphokines such as interleukin-2 [50]. Although it was originally used to suppress rejection following solid organ transplantation, its ophthalmologic use is well established. It has been found to be effective in the treatment of Behçet's disease, VKH, sarcoidosis, sympathetic ophthalmia and birdshot retinochoroidopathy [51, 52].

Cyclosporine is administered orally with an initial dose of 2.5 mg/kg/day and increased in increments of 50 mg to a maximum dose of 5 mg/kg/day based on clinical response [24]. Side effects include hypertension, renal toxicity, hypertrichosis, gingival hyperplasia, myalgia, tremor, paresthesia, and lymphoma. Side effects are more likely to occur with doses higher than 10 mg/kg/day or with prolonged use. Baseline labs should include CBC with differential, blood electrolytes with BUN and creatinine, LFTs, urinalysis and blood pressure. Blood pressure and blood electrolytes with BUN are monitored every 2 weeks and then monthly along with liver function tests, creatinine, and CBC once therapy is stabilized [30]. Treatment is continued for 2 years following ocular quiescence [24]. 

#### 3.3.2. Tacrolimus (FK506)

Tacrolimus (Prograf, Astellas, Tokyo, Japan) is a macrolide antibiotic that like cyclosporine, inhibits calcineurin and suppresses T-cell signal transduction and interleukin-2 transcription [53]. Although clinical experience for treatment of uveitis is more limited than that of cyclosporine, tacrolimus has been found to be effective in the treatment of intraocular inflammation usually in conjunction with systemic corticosteroids [54]. It has also been found to be effective in treatment failure with cyclosporine [55, 56].

Tacrolimus is administered orally at a dose of 0.10 to 0.15 mg/kg/day. It is also available for intravenous use. Adverse events are similar to those seen with cyclosporine. In addition to hypertension and nephrotoxicity, side effects include hyperglycemia, hyperkalemia, hypomagnesemia, loss of appetite and neurologic symptoms such as insomnia, confusion, depression, catatonia, tremors and seizures, and increased risk of non-Hodgkin's lymphoma. The more severe symptoms are seen at higher doses when given intravenously in transplant patients [57–59]. Laboratory evaluation is similar to that for cyclosporine and includes baseline CBC, LFTs, and electrolytes with BUN and creatinine. Blood pressure and blood electrolytes with BUN are monitored every 2 weeks and then monthly along with liver function tests, creatinine, and CBC once therapy is stabilized.

#### 3.3.3. Rapamycin (Sirolimus)

Rapamycin (Rapamune, Phizer, New York, NY USA) is a macrolide antibiotic that inhibits cellular response to interleukin-2 to block B and T lymphocyte activation. Unlike tacrolimus and cyclosporine, rapamycin inhibits the mammalian target of rapamycin (mTOR) and is not a calcineurin inhibitor. Thus it is thought to be less nephrotoxic. Early studies suggest that rapamycin may be most useful in combination with other immunosuppressive agents [60, 61].

Rapamycin is administered orally at a loading dose of 6 mg followed by a daily dose of 2 to 6 mg/day [60]. Baseline and follow-up laboratory and blood pressure monitoring is similar to that of cyclosporine and tacrolimus. Described side effects include elevated liver enzymes, thrombocytopenia, anemia, hypercholesterolemia, nausea, abdominal pain and eczema. All high-dose immunosuppressants given to transplant patients carry an elevated risk of malignancy, and the risk to uveitis patients is likely proportional to dose and duration.

### 3.4. Biologic Agents

Biologic response modifiers, also known as biologics, are a newer class of therapeutic proteins used to treat uveitis by inhibiting bioactive mediators or cytokines such as tumor necrosis factor alpha (TNF-*α*) and interleukin-2. These agents were developed for the treatment of systemic inflammatory diseases and for the prevention of solid organ transplant failure. They target specific molecules in the inflammatory process and may be an alternative in treating uveitis refractory to conventional treatment. Because of the integral immunologic role of TNF, its suppression increases the risk of latent and opportunistic infections such as tuberculosis, histoplasmosis, coccidiomycosis, and herpes viruses. In addition, there are rare reports of lymphoma and other malignancies with the use of TNF blockers. Use of biologics for the treatment of uveitis is considered off-label in the USA.

There are 2 major groups of biologic agents, monoclonal antibodies and fusion proteins. Types of monoclonal antibodies are identified by their suffix. Antibody sequences are human if the suffix is “-umab,” human-murine (human constant and murine variable regions) if the suffix is “-ximab” and humanized (human constant and murine/human variable regions) if the suffix is “-zumab.” Fusion proteins are created by joining two or more genes originally coded for separate proteins. They are composed of a receptor with specificity to the molecule of interest fused with another protein fragment such as a portion of an antibody. The suffix ending in “-cept” denotes fusion proteins. 

#### 3.4.1. Etanercept

Etanercept (Enbrel, Pfizer, New York, NY USA) is a fusion protein composed of a TNF receptor and the Fc fragment of human IgG antibody. It inhibits the binding of TNF-*α* and TNF-*β* to the surface TNF receptors, inactivating TNF and suppressing neutrophil migration and pro-inflammatory cytokine synthesis. Clinical studies have been indeterminate regarding the efficacy of etanercept for the treatment of ocular inflammation [62–65]. 

Etanercept is administered subcutaneously, 25 mg twice a week for 2 years. Contraindications to Etanercept include a history of latent tuberculosis (TB) and exposure to hepatitis B. Adverse effects of etanercept include infection, reactivation of latent TB and hepatitis B, and rare reports of pancytopenia, central nervous system demyelination, congestive heart failure, and lymphoma [66, 67]. Tuberculin skin testing and hepatitis B serologic testing are performed at the time of screening. CBC and LFTs are also performed at baseline and repeated monthly [30, 68].

#### 3.4.2. Infliximab

Infliximab (Remicade, Janssen, Beerse, Belgium) is a monoclonal antibody that binds and inhibits both bound and circulating TNF-*α* [69]. It has shown encouraging responses in patients with treatment-resistant ocular inflammation including Behçet's disease, Wegener's granulomatosis, sarcoidosis, and juvenile inflammatory arthritis [70–74].

Infliximab is administered intravenously with loading infusions at weeks 0, 2, and 6. The doses are 5 mg/kg for monotherapy and 3 mg/kg in patients receiving concurrent noncorticosteroid immunomodulatory treatment. Maintenance infusions are then performed every 8 weeks [74]. Adverse events include infections including upper respiratory and urinary tract, cough, rash, nausea, vomiting, abdominal pain, headache, lupus-like illness, vasculitis, anemia, and thrombocytopenia [74–76]. Tuberculin skin testing is performed at the time of screening. CBC and LFTs are also performed at baseline and repeated monthly. Treatment is maintained for 2 years after ocular quiescence is achieved [17]. 

#### 3.4.3. Adalimumab

Adalimumab (Humira, Abbott) is a recombinant humanized monoclonal antibody that binds and inhibits TNF-*α* [77]. Adalimumab has been used with increasing frequency and found to be effective for treatment of Behçet's disease, VKH, birdshot retinochoroidopathy, juvenile inflammatory arthritis, and scleritis due to rheumatoid arthritis [78–82]. 

Adalimumab is administered subcutaneously at a dose of 40 mg every two weeks [83]. Adverse effects are similar to those of infliximab and etanercept and include the reactivation of latent infections such as tuberculosis and opportunistic infections. The most common side effects are injection site reactions, upper respiratory and urinary tract infections, headache, confusion and rare reports of central nervous system demyelination, hepatotoxicity, congestive heart failure and lymphoma [84, 85]. As with other TNF-*α* inhibitors, tuberculin skin testing is performed at the time of screening. CBC and LFTs are also performed at baseline and repeated monthly. Treatment is maintained for 2 years after ocular quiescence is achieved [17].

#### 3.4.4. Daclizumab

Daclizumab (Zenapax, Genentech/Roche) is a humanized monoclonal antibody to the interleukin-2 receptor on T lymphocytes [86]. In several small case series, daclizumab has been found useful in treating birdshot retinochoroidopathy, posterior uveitis and juvenile inflammatory arthritic uveitis [87–89].Daclizumab, which was discontinued by Hoffman La Roche on Sept 01, 2009, is no longer available to the US market and is primarily used through participation in clinical trials.

Daclizumab is administered intravenously at 1 mg/kg every 2 weeks with or without other immunomodulators. The dose is then adjusted based on the clinical response to a maximum daily dose of 200 mg [90]. Daclizumab is generally better tolerated than TNF-*α* inhibitors. Adverse effects include rash, lymphadenopathy, chest discomfort, and fever [91]. Baseline laboratory evaluation includes CBC and LFTs, which are repeated prior to each infusion. Treatment is maintained for 2 years after ocular quiescence is achieved [87]. 

#### 3.4.5. Rituximab

Rituximab (Rituxan, Biogen Idec, Weston, MA) is a chimeric monoclonal antibody that binds to CD20 antigen on the surface of B cells and suppresses B-cell differentiation resulting in reduced IgG and IgM production [92]. It has been found to be effective in treatment of Behçet's disease, Wegener's granulomatosis uveitis and retinal vasculitis [93, 94]. It has also been used in conjunction with intravenous IgG in the treatment of ocular cicatricial pemphigoid [95]. It is important to emphasize that rituximab treatment is associated with a risk of death from severe side effects, such as *pneumocystis* infection, toxic epidermal necrolysis, and progressive multifocal leukoencephalopathy [96, 97]. Other adverse events include severe infusion reaction, infection, and acute renal failure [98]. 

#### 3.4.6. Tocilizumab

Tocilizumab (Actemra, Roche, Basel, Switzerland) is a humanized monoclonal antibody against the interleukin-6 (IL-6) receptors on T-and B-cells and monocytes and hinders IL-6 expression. It is primarily used for the treatment of rheumatoid arthritis and received FDA approval in April 2011 for treatment of systemic juvenile idiopathic arthritis [99]. IL-6 has been found to be elevated in the vitreous of patients with active posterior uveitis [100]. Tocilizumab for ophthalmic use has been limited to date. It has recently shown promise in the treatment of refractory uveitis [101].

Common side effects include upper respiratory tract infections, nasopharyngitis, hypertension, headache, and transient increases in alanine transaminase [102]. Less common side effects include neutropenia, thrombocytopenia gastritis, gastrointestinal perforations, and opportunistic or recurrent infections such as tuberculosis and fungal infections [103]. 

### 3.5. Other

#### 3.5.1. Interferons

Interferons (IFNs) are endogenous cytokines that are released by a variety of cells in response to the presence of external pathogens such as viruses, bacteria, and tumor cells. There are multiple classes of interferons but IFN-*α* 2a and 2b and IFN-*β* 1a and 1b are used therapeutically for high-risk cutaneous melanoma, hepatitis C, and multiple sclerosis [104, 105]. IFN-*α* 2a has been used successfully in the treatment of Behçet's disease [106, 107]. IFN-*β* 1a has been used to reduce recurrences of uveitis in patients with multiple sclerosis [108, 109].

IFN-*α* 2a is typically administered at a dose of 3 to 6 million international units each day to 3 times weekly [110]. The most common adverse effects include fever, chills, myalgias, fatigue, alopecia, and depression [111]. Interferon retinopathy has been reported and should be evaluated. Baseline laboratory tests include CBC, LFTs and thyroid function. CBC and LFTs are repeated at 4 week intervals, while thyroid function tests are performed every 3 months. The goal of treatment is to achieve ocular inflammatory quiescence for 2 years before stopping therapy [6]. 

## 4. Conclusion

In summary, a heterogeneous group of non-infectious inflammatory diseases result in vision-threatening vitreous inflammation. The goal of treatment is to eliminate intraocular inflammation rapidly while closely monitoring drug side effects. There are now many classes of drugs which may be used as monotherapy or in combination to achieve this goal. Many of these inflammatory disorders require long-term steroid-sparing agents to adequately control disease, usually beyond two years. Immunomodulatory therapy requires close monitoring due to potential adverse effects and varied individual responses. Biologic agents may be an alternative for patients with refractory uveitis. Further studies are required to determine their efficacy. 

## Figures and Tables

**Table 1:** Diseases indicated for immunosuppressive chemotherapy.

Strong indications	Relative indications	Questionable indications
Behçet's disease with retinal involvement	Intermediate uveitis	Intermediate uveitis in children
Sympathetic ophthalmia	Retinal vasculitis with central vascular leakage	Sarcoid-associated uveitis inadequately responsive to steroid
Vogt-Koyanagi-Harada syndrome	Severe chronic iridocyclitis	Keratoplasty with multiple rejections
Rheumatoid necrotizing scleritis or peripheral ulcerative keratitis		
Wegener's granulomatosis		
Relapsing polychondritis with scleritis		
Juvenile idiopathic arthritis-associated iridocyclitis unresponsive to conventional therapy		
Ocular cicatricial pemphigoid		
Bilateral Mooren ulcer		

**Table 2:** Uses of immunosuppressive agents in ophthalmologic conditions.

Drug	Diagnosis
Antimetabolites	
Methotrexate	Chronic non-infectious uveitis, sarcoidosis, and non-infectious ocular inflammation
Azathioprine	Chronic uveitis, Behcet's disease, choroidal neovascularization, OCP, retinal vasculitis, serpiginous choroiditis, and neuroretinitis
Mycophenolate mofetil	Chronic uveitis, non-infectious ocular inflammation, intermediate and posterior uveitis, refractory uveitis, and scleritis
Leflunomide	Sarcoidosis uveitis

Alkylating agents	
Cyclophosphamide	Refractory uveitis, peripheral uveitis, Wegener, OCP, scleritis, Behçet's disease, non-infectious ocular inflammation, and optic neuropathy (SLE)
Chlorambucil	Serpiginous choroiditis, severe chronic uveitis, uveitis, and Behçet's disease

T-cell inhibitors/calcineurin inhibitors	
Cyclosporine	Serpiginous choroidopathy, Behçet's disease, endogenous uveitis, chronic idiopathic uveitis, scleritis, rheumatoid arthritis, and non-infectious uveitis
Tacrolimus	Refractory uveitis (limited experience)
Rapamycin	Refractory uveitis (limited experience)

Biologic agents	
Etanercept	Juvenile idiopathic arthritis, non-infectious uveitis, and ocular inflammatory disease
Infliximab	Refractory uveitis, childhood uveitis, Behcet's disease, and refractory uveitis
Adalimumab	Refractory uveitis, ankylosing spondylitis, and juvenile idiopathic arthritis
Daclizumab	Juvenile idiopathic arthritis, recalcitrant ocular inflammation, and birdshot retinochoroidopathy
Rituximab	Primary Sjogren syndrome, thyroid eye disease, and Wegener
Tocilizumab	Refractory uveitis (limited experience)

Other	
Interferons	Behcet's disease, non-infectious uveitis

## References

[1] Foster CS, Vitale AT (2002). *Diagnosis and Treatment of Uveitis*.

[2] Jabs DA, Akpek EK (2005). Immunosuppression for posterior uveitis. *Retina*.

[3] Kovacs K, Wagley S, Quirk MT (2012). Pharmacokinetic study of vitreous and serum concentrations of triamcinolone acetonide after posterior sub-tenon’s injection. *American Journal of Ophthalmology*.

[4] Albert DM, Jakobiec FA (2000). *Principles and Practice of Ophthalmology*.

[5] Sabrosa NA, Pavésio C (2000). Treatment strategies in patients with posterior uveitis. *International Ophthalmology Clinics*.

[6] Durrani K, Zakka FR, Ahmed M, Memon M, Siddique SS, Foster CS (2011). Systemic therapy with conventional and novel immunomodulatory agents for ocular inflammatory disease. *Survey of Ophthalmology*.

[7] Okada AA (2005). Immunomodulatory therapy for ocular inflammatory disease: a basic manual and review of the literature. *Ocular Immunology and Inflammation*.

[8] Zimmerman TJ (1997). *Textbook of Ocular Pharmacology*.

[9] Bom S, Zamiri P, Lightman S (2001). Use of methotrexate in the management of sight-threatening uveitis. *Ocular Immunology and Inflammation*.

[10] Holz FG, Krastel H, Breitbart A, Schwarz-Eywill M, Pezzutto A, Völcker HE (1992). Low-dose methotrexate treatment in noninfectious uveitis resistant to corticosteroids.. *German Journal of Ophthalmology*.

[11] Kaplan-Messas A, Barkana Y, Avni I, Neumann R (2003). Methotrexate as a first-line corticosteroid-sparing therapy in a cohort of uveitis and scleritis. *Ocular Immunology and Inflammation*.

[12] Shah SS, Lowder CY, Schmitt MA, Wilke WS, Kosmorsky GS, Meisler DM (1992). Low-dose methotrexate therapy for ocular inflammatory disease. *Ophthalmology*.

[13] Gangaputra S, Newcomb CW, Liesegang TL (2009). Methotrexate for ocular inflammatory diseases. *Ophthalmology*.

[14] Samson CM, Waheed N, Baltatzis S, Foster CS (2001). Methotrexate therapy for chronic noninfectious uveitis: analysis of a case series of 160 patients. *Ophthalmology*.

[15] Weinblatt ME (1985). Toxicity of low dose methotrexate in rheumatoid arthritis. *Journal of Rheumatology*.

[16] BenEzra D, Cohen E (2000). Cataract surgery in children with chronic uveitis. *Ophthalmology*.

[17] Lee FF, Foster CS (2010). Pharmacotherapy of uveitis. *Expert Opinion on Pharmacotherapy*.

[18] Elion GB (1977). Pharmacologic and physical agents. Immunosuppressive agents.. *Transplantation Proceedings*.

[19] Chan GLC, Canafax DM, Johnson CA (1987). The therapeutic use of azathioprine in renal transplantation. *Pharmacotherapy*.

[20] Hooper PL, Kaplan HJ (1991). Triple agent immunosuppression in serpiginous choroiditis. *Ophthalmology*.

[21] Michel SS, Ekong A, Baltatzis S, Foster CS (2002). Multifocal choroiditis and panuveitis: immunomodulatory therapy. *Ophthalmology*.

[22] Saw VPJ, Dart JKG, Rauz S (2008). Immunosuppressive therapy for ocular mucous membrane pemphigoid. Strategies and outcomes. *Ophthalmology*.

[23] Whisnant JK, Pelkey J (1982). Rheumatoid arthritis: treatment with azathioprine (IMURAN (R)). Clinical side-effects and laboratory abnormalities. *Annals of the Rheumatic Diseases*.

[24] Foster CS, Vitale AT, Foster CS, Vitale AT (2002). Immunosuppressive chemotherapy. *Diagnosis and Treatment of Uveitis*.

[25] Vavvas D, Foster CS (2004). Immunomodulatory medications in uveitis. *International Ophthalmology Clinics*.

[26] Allison AC, Eugui EM (1993). Immunosuppressive and other effects of mycophenolic acid and an ester prodrug, mycophenolate mofetil. *Immunological Reviews*.

[27] Voisard R, Viola S, Kaspar V (2005). Effects of mycophenolate mofetil on key pattern of coronary restenosis: a cascade of in vitro and ex vivo models. *BMC Cardiovascular Disorders*.

[28] Baltatzis S, Tufail F, Yu EN, Vredeveld CM, Foster CS (2003). Mycophenolate mofetil as an immunomodulatory agent in the treatment of chronic ocular inflammatory disorders. *Ophthalmology*.

[29] Sobrin L, Christen W, Foster CS (2008). Mycophenolate mofetil after methotrexate failure or intolerance in the treatment of scleritis and uveitis. *Ophthalmology*.

[30] Jabs DA, Rosenbaum JT, Foster CS (2000). Guidelines for the use of immunosuppressive drugs in patients with ocular inflammatory disorders: recommendations of an expert panel. *American Journal of Ophthalmology*.

[31] Fox RI, Herrmann ML, Frangou CG (1999). Mechanism of action for leflunomide in rheumatoid arthritis. *Clinical Immunology*.

[32] Silva HT, Morris RE (1997). Leflunomide and malononitriloamides. *Expert Opinion on Investigational Drugs*.

[33] Anon

[34] Baughman RP, Lower EE (2004). Leflunomide for chronic sarcoidosis. *Sarcoidosis Vasculitis and Diffuse Lung Diseases*.

[35] Roussel HM (1999). *Summary of Product Characteristics for Arava*.

[36] Cohen S, Cannon GW, Schiff M (2001). Two-year, blinded, randomized, controlled trial of treatment of active rheumatoid arthritis with leflunomide compared with methotrexate. *Arthritis and Rheumatism*.

[37] Fauci AS, Wolff SM, Johnson JS (1971). Effect of cyclophosphamide upon the immune response in Wegener’s granulomatosis.. *New England Journal of Medicine*.

[38] Buckley CE, Gills JP (1969). Cyclophosphamide therapy of Behcet’s disease. *Journal of Allergy*.

[39] Foster CS, Wilson LA, Ekins MB (1982). Immunosuppressive therapy for progressive ocular cicatricial pemphigoid. *Ophthalmology*.

[40] Fosdick WM, Parsons JL, Hill DF (1968). Long-term cyclophosphamide therapy in rheumatoid arthritis.. *Arthritis and Rheumatism*.

[41] Brubaker R, Font RL, Shepherd EM (1971). Granulomatous sclerouveitis. Regression of ocular lesions with cyclophosphamide and prednisone. *Archives of Ophthalmology*.

[42] Fauci AS, Doppman JL, Wolff SM (1978). Cyclophosphamide-induced remissions in advanced polyarteritis nodosa. *American Journal of Medicine*.

[43] Hoang-Xuan T, Foster CS, Rice BA (1990). Scleritis in relapsing polychondritis. Response to therapy. *Ophthalmology*.

[44] Durrani K, Papaliodis GN, Foster CS (2004). Pulse IV cyclophosphamide in ocular inflammatory disease: efficacy and short-term safety. *Ophthalmology*.

[45] Chabner BA, Amrein PC, Druker B, Goodman LS, Gilman A, Brunton LL, Lazo JS, Parker KL (2006). Antineoplastic agents. *Goodman & Gilman’s the Pharmacological Basis of Therapeutics*.

[46] Miserocchi E, Baltatzis S, Ekong A, Roque M, Foster CS (2002). Efficacy and safety of chlorambucil in intractable noninfectious uveitis: the Massachusetts eye and ear infirmary experience. *Ophthalmology*.

[47] Mudun AB, Ergen A, Ipcioglu SU, Yarkin Burumcek E, Durlu Y, Okan Arslan M (2001). Short-term chlorambucil for refractory uveitis in Behcet’s disease. *Ocular Immunology and Inflammation*.

[48] Cannon GW, Jackson CG, Samuelson CO (1985). Chlorambucil therapy in rheumatoid arthritis: clinical experience in 28 patients and literature review. *Seminars in Arthritis and Rheumatism*.

[49] Tabbara KF (1983). Chlorambucil in Behcet’s disease. A reappraisal. *Ophthalmology*.

[50] Gerber DA, Bonham CA, Thomson AW (1998). Immunosuppressive agents: recent developments in molecular action and clinical application. *Transplantation Proceedings*.

[51] Palestine AG, Nussenblatt RB, Gelato M (1988). Therapy for human autoimmune uveitis with low-dose cyclosporine plus bromocriptine. *Transplantation Proceedings*.

[52] Vitale AT, Rodriguez A, Foster CS (1994). Low-dose cyclosporine therapy in the treatment of birdshot retinochoroidopathy. *Ophthalmology*.

[53] Liu J, Farmer JD, Lane WS, Friedman J, Weissman I, Schreiber SL (1991). Calcineurin is a common target of cyclophilin-cyclosporin A and FKBP-FK506 complexes. *Cell*.

[54] Mochizuki M, Masuda K, Sakane T (1993). A clinical trial of FK506 in refractory uveitis. *American Journal of Ophthalmology*.

[55] Kilmartin DJ, Forrester JV, Dick AD (1998). Tacrolimus (FK506) in failed cyclosporin A therapy in endogenous posterior uveitis. *Ocular Immunology and Inflammation*.

[56] Sloper CML, Powell RJ, Dua HS (1999). Tacrolimus (FK506) in the treatment of posterior uveitis refractory to cyclosporine. *Ophthalmology*.

[57] Naesens M, Kuypers DRJ, Sarwal M (2009). Calcineurin inhibitor nephrotoxicity. *Clinical Journal of the American Society of Nephrology*.

[58] Miwa Y, Isozaki T, Wakabayashi K (2008). Tacrolimus-induced lung injury in a rheumatoid arthritis patient with interstitial pneumonitis. *Modern Rheumatology*.

[59] O’Donnell MM, Williams JP, Weinrieb R, Denysenko L (2007). Catatonic mutism after liver transplant rapidly reversed with lorazepam. *General Hospital Psychiatry*.

[60] Shanmuganathan VA, Casely EM, Raj D (2005). The efficacy of sirolimus in the treatment of patients with refractory uveitis. *British Journal of Ophthalmology*.

[61] Phillips BN, Wroblewski KJ (2011). A retrospective review of oral low-dose sirolimus (rapamycin) for the treatment of active uveitis. *Journal of Ophthalmic Inflammation and Infection*.

[62] Reiff A (2003). Long-term outcome of etanercept therapy in children with treatment-refractory uveitis. *Arthritis and Rheumatism*.

[63] Sfikakis PP (2002). Behçet’s disease: a new target for anti-tumour necrosis factor treatment. *Annals of the Rheumatic Diseases*.

[64] Lim LL, Fraunfelder FW, Rosenbaum JT (2007). Do tumor necrosis factor inhibitors cause uveitis? A registry-based study. *Arthritis and Rheumatism*.

[65] Galor A, Perez VL, Hammel JP, Lowder CY (2006). Differential effectiveness of etanercept and infliximab in the treatment of ocular inflammation. *Ophthalmology*.

[66] Desai SB, Furst DE (2006). Problems encountered during anti-tumour necrosis factor therapy. *Best Practice and Research*.

[67] Scheinfeld N (2004). A comprehensive review and evaluation of the side effects of the tumor necrosis factor alpha blockers etanercept, infliximab and adalimumab. *Journal of Dermatological Treatment*.

[68] Lin J, Ziring D, Desai S (2008). TNFalpha blockade in human diseases: an overview of efficacy and safety. *Clinical Immunology*.

[69] Calabrese LH (2003). Molecular differences in anticytokine therapies. *Clinical and Experimental Rheumatology*.

[70] Bodaghi B, Bui Quoc E, Wechsler B (2005). Therapeutic use of infliximab in sight threatening uveitis: retrospective analysis of efficacy, safety, and limiting factors. *Annals of the Rheumatic Diseases*.

[71] Baughman RP, Bradley DA, Lower EE (2005). Infliximab in chronic ocular inflammation. *International Journal of Clinical Pharmacology and Therapeutics*.

[72] Kahn P, Weiss M, Imundo LF, Levy DM (2006). Favorable response to high-dose infliximab for refractory childhood uveitis. *Ophthalmology*.

[73] Niccoli L, Nannini C, Benucci M (2007). Long-term efficacy of infliximab in refractory posterior uveitis of Behçet’s disease: a 24-month follow-up study. *Rheumatology*.

[74] Suhler EB, Smith JR, Giles TR (2009). Infliximab therapy for refractory uveitis: 2-Year results of a prospective trial. *Archives of Ophthalmology*.

[75] Braun J, Brandt J, Listing J (2003). Long-term efficacy and safety of infliximab in the treatment of ankylosing spondylitis: an open, observational, extension study of a three-month, randomized, placebo-controlled trial. *Arthritis and Rheumatism*.

[76] Gómez-Reino JJ, Carmona L, Rodríguez Valverde V, Mola EM, Montero MD (2003). Treatment of rheumatoid arthritis with tumor necrosis factor inhibitors may predispose to significant increase in tuberculosis risk: a multicenter active-surveillance report. *Arthritis and Rheumatism*.

[77] Kaymakcalan Z, Sakorafas P, Bose S (2009). Comparisons of affinities, avidities, and complement activation of adalimumab, infliximab, and etanercept in binding to soluble and membrane tumor necrosis factor. *Clinical Immunology*.

[78] Mushtaq B, Saeed T, Situnayake RD, Murray PI (2007). Adalimumab for sight-threatening uveitis in Behçet’s disease. *Eye*.

[79] Diaz-Llopis M, García-Delpech S, Salom D (2008). Adalimumab therapy for refractory uveitis: a pilot study. *Journal of Ocular Pharmacology and Therapeutics*.

[80] Restrepo JP, Molina MP (2010). Successful treatment of severe nodular scleritis with adalimumab. *Clinical Rheumatology*.

[81] Vazquez-Cobian LB, Flynn T, Lehman TJA (2006). Adalimumab therapy for childhood uveitis. *Journal of Pediatrics*.

[82] Tynjäaldie; P, Kotaniemi K, Lindahl P (2008). Adalimumab in juvenile idiopathic arthritis-associated chronic anterior uveitis. *Rheumatology*.

[83] Rudwaleit M, Rødevand E, Holck P (2009). Adalimumab effectively reduces the rate of anterior uveitis flares in patients with active ankylosing spondylitis: results of a prospective open-label study. *Annals of the Rheumatic Diseases*.

[84] Singh JA, Wells GA, Christensen R (2011). Adverse effects of biologics: a network meta-analysis and Cochrane overview. *Cochrane Database of Systematic Reviews*.

[85] Alonso-Ruiz A, Pijoan JI, Ansuategui E, Urkaregi A, Calabozo M, Quintana A (2008). Tumor necrosis factor alpha drugs in rheumatoid arthritis: systematic review and metaanalysis of efficacy and safety. *BMC Musculoskeletal Disorders*.

[86] Yang H, Wang J, Du J (2010). Structural basis of immunosuppression by the therapeutic antibody daclizumab. *Cell Research*.

[87] Sobrin L, Huang JJ, Christen W, Kafkala C, Choopong P, Foster CS (2008). Daclizumab for treatment of birdshot chorioretinopathy. *Archives of Ophthalmology*.

[88] Sen HN, Levy-Clarke G, Faia LJ (2009). High-dose daclizumab for the treatment of juvenile idiopathic arthritis-associated active anterior uveitis. *American Journal of Ophthalmology*.

[89] Gallagher M, Quinones K, Cervantes-Castañeda RA, Yilmaz T, Foster CS (2007). Biological response modifier therapy for refractory childhood uveitis. *British Journal of Ophthalmology*.

[90] Bhat P, Castañeda-Cervantes RA, Doctor PP, Foster CS (2009). Intravenous daclizumab for recalcitrant ocular inflammatory disease. *Graefe’s Archive for Clinical and Experimental Ophthalmology*.

[91] Rojas MA, Carlson NG, Miller TL, Rose JW (2009). Long-term daclizumab therapy in relapsing-remitting multiple sclerosis. *Therapeutic Advances in Neurological Disorders*.

[92] Lim L, Suhler EB, Smith JR (2006). Biologic therapies for inflammatory eye disease. *Clinical and Experimental Ophthalmology*.

[93] Davatchi F, Shams H, Rezaipoor M (2010). Rituximab in intractable ocular lesions of Behcet’s disease; randomized single-blind control study (pilot study). *International Journal of Rheumatic Diseases*.

[94] Taylor SRJ, Salama AD, Joshi L, Pusey CD, Lightman SL (2009). Rituximab is effective in the treatment of refractory ophthalmic Wegener’s granulomatosis. *Arthritis and Rheumatism*.

[95] Foster CS, Chang PY, Ahmed AR (2010). Combination of rituximab and intravenous immunoglobulin for recalcitrant ocular cicatricial pemphigoid. A preliminary report. *Ophthalmology*.

[96] Bermudez A, Marco F, Conde E, Mazo E, Recio M, Zubizarreta A (2000). Fatal visceral varicella-zoster infection following rituximab and chemotherapy treatment in a patient with follicular lymphoma. *Haematologica*.

[97] Quartier P, Tournilhac O, Archimbaud C (2003). Enteroviral meningoencephalitis after anti-CD20 (rituximab) treatment.. *Clinical Infectious Diseases*.

[98] Genentech

[99] Jones G, Sebba A, Gu J (2010). Comparison of tocilizumab monotherapy versus methotrexate monotherapy in patients with moderate to severe rheumatoid arthritis: the AMBITION study. *Annals of the Rheumatic Diseases*.

[100] Perez VL, Papaliodis GN, Chu D, Anzaar F, Christen W, Foster CS (2004). Elevated levels of interleukin 6 in the vitreous fluid of patients with pars planitis and posterior uveitis: the Massachusetts eye & ear experience and review of previous studies. *Ocular Immunology and Inflammation*.

[101] Muselier A, Bielefeld P, Bidot S, Vinit J, Besancenot J-F, Bron A (2011). Efficacy of tocilizumab in two patients with anti-TNF-alpha refractory uveitis. *Ocular Immunology and Inflammation*.

[102] Dinnendahl V, Fricke U (2010). *Arzneistoff-Profile*.

[103] Oldfield V, Dhillon S, Plosker GL (2009). Tocilizumab a review of its use in the management of rheumatoid arthritis. *Drugs*.

[104] Hayden F, Goodman LS, Gilman A, Brunton LL, Lazo JS, Parker KL (2006). Antiviral agents (non-retroviral). *Goodman & Gilman’s the Pharmacological Basis of Therapeutics*.

[105] Mocellin S, Pasquali S, Rossi CR, Nitti D (2010). Interferon alpha adjuvant therapy in patients with high-risk melanoma: a systematic review and meta-analysis. *Journal of the National Cancer Institute*.

[106] Alpsoy E, Durusoy C, Yilmaz E (2002). Interferon alfa-2a in the treatment of Behçet disease: a randomized placebo-controlled and double-blind study. *Archives of Dermatology*.

[107] Kotter I, Vonthein R, Zierhut M (2004). Differential efficacy of human recombinant interferon-alpha2a on ocular and extraocular manifestations of Behcet disease: results of an open 4-center trial. *Seminars in Arthritis and Rheumatism*.

[108] Mackensen F, Max R, Becker MD (2009). Interferons and their potential in the treatment of ocular inflammation. *Clinical Ophthalmology*.

[109] Becker MD, Heiligenhaus A, Hudde T (2005). Interferon as a treatment for uveitis associated with multiple sclerosis. *British Journal of Ophthalmology*.

[110] Deuter CME, Zierhut M, Möhle A, Vonthein R, Stübiger N, Kötter I (2010). Long-term remission after cessation of interferon-*α* treatment in patients with severe uveitis due to Behçet’s disease. *Arthritis and Rheumatism*.

[111] Kotter I, Günaydin I, Zierhut M, Stübiger N (2004). The use of interferon alpha in Behcet disease: review of the literature. *Seminars in Arthritis and Rheumatism*.

